# Miss Blackwell, M. D.

**Published:** 1849-10

**Authors:** 


### MISS BLACKWELL, M. D.

We learn from the Buffalo Medical Journal, that Miss Blackwell, the female graduate of Geneva College, is now, with praiseworthy assiduity, prosecuting her professional studies at the *Hopital de Maternite*, in Paris.

